# Early diagnosis of anastomotic leakage after colorectal cancer surgery using an inflammatory factors-based score system

**DOI:** 10.1093/bjsopen/zrac069

**Published:** 2022-06-03

**Authors:** Jinyao Shi, Zhouqiao Wu, Xiaolong Wu, Fei Shan, Yan Zhang, Xiangji Ying, Ziyu Li, Jiafu Ji

**Affiliations:** Gastrointestinal Cancer Center, Key Laboratory of Carcinogenesis and Translational Research (Ministry of Education), Peking University Cancer Hospital & Institute, Beijing, P.R. China

## Abstract

**Background:**

Anastomotic leakage (AL) is a severe complication after colorectal surgery. This study aimed to investigate a method for the early diagnosis of AL after surgical resection by analysing inflammatory factors (IFs) in peritoneal drainage fluid.

**Methods:**

Abdominal drainage fluid of patients with colorectal cancer who underwent resection between April 2017 and April 2018, were prospectively collected in the postoperative interval. Six IFs, including interleukin (IL)-1β, IL-6, IL-10, tumour necrosis factor (TNF)-α, matrix metalloproteinase (MMP)2, and MMP9, in drainage were determined by multiplex immunoassay to investigate AL (in patients undergoing resection and anastomosis) and pelvic collection (in patients undergoing abdominoperineal resection). Sparreboom and colleagues’ prediction model was first evaluated for AL/pelvic collection, followed by a new IF-based score system (AScore) that was developed by a least absolute shrinkage and selection operator (LASSO) regression, for the same outcomes. The model performance was tested for the area under the curve (AUC), sensitivity, specificity, negative predictive value (NPV), and positive predictive value (PPV).

**Results:**

Out of 123 patients eligible, 119 patients were selected, including 12 patients with AL/pelvic collection. Sparreboom and colleagues’ prediction model was documented with the best diagnostic efficacy on postoperative day 3 (POD3), with an AUC of 0.77. After optimization, AScore on POD3 increased the AUC to 0.83 and on POD1 showed the best diagnostic efficiency, with an AUC of 0.88. Based on the Youden index, the cut-off value of AScore on POD1 was set as −2.46 to stratify patients into low-risk and high-risk groups for AL/pelvic collection. The model showed 90.0 per cent sensitivity, 69.7 per cent specificity, 98.4 per cent NPV, and 25.0 per cent PPV.

**Conclusions:**

The early determination of IFs in abdominal drainage fluid of patients undergoing colorectal surgery could be useful to predict AL or pelvic collection.

## Teaser

Anastomotic leakage can be diagnosed at postoperative day 1 after colorectal surgery by analysing inflammatory factors from peritoneal drainage.

## Introduction

Anastomotic leakage (AL) is a severe complication after colorectal surgery that may cause intra-abdominal infection and sepsis, and a prolonged duration of hospital stay; also, it has been correlated with higher rates of local tumour recurrence^1,2^. Literature documented an incidence of AL ranging between 3–19 per cent^3–5^ after resection; however, its early identification remains an issue.

Indeed, although the clinical management of patients with colorectal cancer has been improved by enhanced recovery after surgery (ERAS) programmes, that allow early recovery and early discharge, the prompt diagnosis of AL may avoid serious complications before discharge and critical readmissions^6^.

C-reactive protein (CRP) is a common indicator used in clinical practice to evaluate inflammatory responses, including those induced by a postoperative AL^7,8^. The PREDICT study^9^, which recruited more than 900 patients from 20 hospitals across four western countries, revealed that CRP achieved the best predictive efficacy for colorectal AL on postoperative day (POD) 5, with an area under the curve (AUC) of 0.79, whereas on POD3, the AUC was approximately 0.67.

In contrast to serum biomarkers, inflammatory factors (IFs), such as cytokines and matrix metalloproteinases (MMPs) in the abdominal drainage, seem to reflect abnormal anastomotic healing more accurately in the early phase. The APPEAL-II trial by Sparreboom and colleagues, established an AL prediction model by combining serum CRP with MMP9 in the abdominal drainage^10^. This model could increase the AUC to 0.78 on POD3, improving the median diagnostic time of AL currently reported at 6 days^11,12^. Therefore, detecting AL by measuring IFs in abdominal drainage may be a promising method to enhance clinical practice.

This study aimed to evaluate a prediction model based on IF collection and determination in abdominal drains in a series of patients who underwent colorectal resection, aiming for early diagnosis of AL or pelvic collection in patients undergoing abdominoperineal resection.

## Methods

### Study design and patients

This study was designed as a prospective cohort study according to TRIPOD guidelines. Patients who underwent surgical resection for colorectal cancer with a primary anastomosis, or abdominoperineal resection, between April 2017 and April 2018 in the Gastrointestinal Cancer Center, Ward I, Peking University Cancer Hospital and Institute, were included. Of note, the institution is a referral centre for colorectal cancer, where 70 per cent of the patients are referred from different hospitals and more than 600 gastrointestinal, including gastric, intestinal, and colorectal surgeries are performed annually.

The detailed inclusion criteria were patients older than 18 years with a diagnosis of colon and rectal cancer at any stage. All included patients accepted colorectal resection with construction of a colorectal or coloanal anastomosis. Moreover, patients with abdominal pelvic resection (APR) were also enrolled for analysis, as pelvic collection after the procedure is a well known source of infection. All included patients signed informed consent. Exclusion criteria were patients without informed consent, no drainage fluid obtained, and patients with benign disease, including inflammatory bowel diseases. The research protocol was approved by the ethical committee of Peking University Cancer Hospital.

This cohort was used first to validate the Sparreboom's prediction model (based on serum CRP and drainage MMP9) and subsequently, to optimize the efficiency of the prediction model to obtain a new score (AScore).

### Surgical procedures

At the institution, anastomoses are routinely constructed with the help of linear staplers (for right colectomy) or circular staplers (for left colon, sigmoid, and rectal resection), and manual suturing for a reinforcement is not routinely conducted. An air-leak test is selectively performed in high-risk cases (ultra-low anastomoses) and protective stoma are routinely performed in patients with ultra-low anastomosis (less than 5 cm) and/or preoperative long-term radiotherapy. Although a standard ERAS programme was not routinely conducted during the inclusion interval, patients were allowed to drink and intake enteral nutritional liquid on POD1. Although drainage is routinely applied for left colon, sigmoid, and rectal resection, it is usually removed when AL and abdominal collection are excluded.

### Drain fluid collection and pre-processing

Sample and data collection methods was performed in accordance with the protocol of the APPEAL study^13,14^. Abdominal drainage was collected on the same day of the surgical procedure (POD0) and in the first three PODs (POD1­–3) following colorectal resection with 10 ml EDTA tubes every morning, and approximately 20 ml per patient per day was collected. The drain fluid samples were then immediately centrifuged (2800*g*, 4°C) for 10 min, after which the supernatant and the precipitate were sub-packed into different cryotubes and stored in a −80°C fridge for the subsequent tests.

### Determination of inflammatory factors in drain fluid

Based on the protocol, six IFs (IL-1β, IL-6, IL-10, TNF-α, MMP2, and MMP9) from the drain fluid were measured via multiplex immunoassay. The cytokines (IL-1β, IL-6, IL-10, and TNF-α) were measured with Millipore’s MILLIPLEX® MAP HSTCMAG-28SK kit and the MMPs (MMP2 and MMP9) were measured with Millipore’s MILLIPLEX® MAP HMMP2MAG-55K kit. The concentrations of IFs were analysed on a Luminex® xMAP® platform.

### Clinical data collection

Patient demographic data (sex, age, BMI, ASA score, diabetes, hypertension, and preoperative treatment), surgical procedure, and pathological information (tumour location and cTNM stage) were collected from a prospectively maintained database. CRP levels on POD3 were retrospectively extracted from the electronic medical record system. Postoperative complications were prospectively registered in the case report form up to POD30. The severity of complications was scored by the Clavien–Dindo classification system. The registration items of complications and their diagnostic criteria were based on the Chinese Expert Consensus for Gastrointestinal Cancer Surgery Postoperative Complications Registration^15^. AL was defined as defect anastomosis; manifested radiological changes after surgery (with or without clinical intervention); colour turbidity, faecal, or other indicative changes observed from in the drain fluid; the peri-anastomotic abscess and angiogenic intra-abdominal infection was also considered as AL. In addition, pelvic collection was defined as an unrelated abdominoperineal infection, abscess, or peritonitis, confirmed by radiology, or reoperation.

In this study, contrast-enhanced X-ray examination, or CT was routinely performed after surgery for evaluating anastomosis healing within 2 weeks after surgery. Evaluation of drainage fluids was performed concurrently with sample collection. The presence of AL/pelvic collection in this study was based on a clinical diagnosis, comprehensively confirmed by clinical symptoms, abnormal drainage, radiological changes, and laboratory tests.

### Outcomes of interest

In this study, AL and pelvic collection after APR were considered altogether to validate the Sparreboom CRP and MMP9-based model on POD3 (first outcome). Based on this, the individual risk of AL/pelvic collection could be estimated by a nomogram, which included serum CRP and drainage MMP9 on POD3. An online calculator was built for their model and is available at https://www.evidencio.com/models/show/1537. Data were analysed with their published model.

The second outcome of interest was to develop a better prediction model based on the diagnostic performance of IFs in POD1–3 to obtain a new score (AScore) for the same outcomes.

### Statistical analysis

Quantitative variables following the Gaussian distribution were reported as mean and s.d., whereas non-Gaussian distribution variables were defined by median and interquartile range (i.q.r.). Qualitative variables were reported as frequencies and percentages. Quantitative variables following a Gaussian distribution were compared with a Student’s *t* test, setting the null hypothesis (H_0_) as a null difference between these groups. Quantitative variables following a non-Gaussian distribution were compared with Mann–Whitney *U* tests, setting H_0_ as the probability of 50 per cent that a randomly drawn member of the first population will exceed a member of the second population. A comparison of qualitative variables was performed with a chi-squared test (H_0_ corresponded to no relationship between the categorical variables).

The experimental study was conducted to test the hypotheses: Sparreboom’s prediction model possessed good stability and reliability; early diagnosis of AL/pelvic collection could be performed in POD1–3 based on IF determination in abdominal drainage fluids.

Based on these, 120 patients would be required to establish an AUC greater than 0.78 (based on Sparreboom’s model) with 95 per cent confidence, 90 per cent power, and assuming a 10 per cent occurrence of AL. For further establishing a new model with expected AUC of 0.85, 70 patients were required.

Using the LASSO regression, IFs with highly predictive values were selected to construct a new score (AScore) system. The calculations were performed with the ‘glmnet’ package of R software. A receiver operating characteristic (ROC) analysis was performed to assess the diagnostic accuracy of predictive features by evaluating the AUC (with an H_0_ set as an AUC equal to 0.5). Cut-off values were decided after ROC analysis based on the maximum of the Youden index (sensitivity + specificity − 1). Sensitivity, specificity, positive predictive value (PPV), and negative predictive value (NPV) were calculated. All statistical analyses were performed with either SPSS^®^ version 24 (IBM, Armonk, New York, USA) or R software version 3.6.1 (R Foundation for Statistical Computing). In all cases, a bilateral *P* < 0.05 was considered statistically significant.

## Results

### Study population

A total of 123 patients were considered eligible for inclusion in this study. Drains were not used in 4 patients, and these were excluded, leaving 119 patients for analysis. Demographic and clinical characteristics of patients are shown in *Table 1*. No patients used steroids during the perioperative interval and the majority were treated with laparoscopy.

**Table 1:** Characteristics of patients with or without anastomotic leakage

	Total (*n* = 119)	Without anastomotic leakage/pelvic collection (*n* = 107)	With anastomotic leakage/pelvic collection* (*n* = 12)	*P*†
**Sex**				0.636
Male	72(60.5)	66(61.7)	6(50.0)	
Female	47(39.5)	41(38.3)	6(50.0)	
**Age, years**				
<65	87(73.1)	78(72.9)	9(75.0)	1.000
≥65	32(26.9)	29(27.1)	3(25.0)	
**BMI (kg/m^2^㎡)**		23.6 (21.5–25.6)	24.4 (21.2–26.2)	0.637
**Diabetes**				0.919
No	103(86.6)	92(86.0)	11(91.7)	
Yes	16(13.4)	15(14.0)	1(8.3)	
**Hypertension**				0.259
No	88(73.9)	77(72.0)	11(91.7)	
Yes	31(26.1)	30(28.0)	1(8.3)	
**cTNM stage**				0.731
I	17(14.3)	14(13.1)	3(25.0)	
II	35(29.4)	31(29.0)	4(33.3)	
III	53(44.5)	49(45.8)	4(33.3)	
IV	3(2.5)	3(2.8)	0(0.0)	
**Missing**	11(9.2)	10(9.3)	1(8.3)	
**Tumour location**				0.095
Colon	28(23.5)	27(25.2)	1(8.3)	
Sigmoid	36(30.3)	34(31.8)	2(16.7)	
Rectal	55(46.2)	46(43.0)	9(75.0)	
**Operative approach**				0.815
Open	41(34.5)	36(33.6)	5(41.7)	
Laparoscopic	78(65.5)	71(66.4)	7(58.3)	
**Resection range**				0.069
Right and transverse resection	23(19.3)	21(19.6)	2(16.7)	
Left and sigmoid resection	36(30.3)	34(31.8)	2(16.7)	
Low anterior resection	46(38.7)	40(37.4)	6(50.0)	
Abdominal pelvic resection	11(9.2)	9(8.4)	2(16.7)	
Others	3(2.5)	3(2.8)	0(0.0)	

* Including anastomotic leakage and fistula-associated abdominal pelvic infection. †Pearson χ test. Values are n (%) unless otherwise indicated.

Within POD30, 25 patients developed postoperative complications. Infectious complications (including AL and surgical incision infection) were diagnosed in 14 patients. Among them, 12 patients had AL (10 for AL and 2 for pelvic collection). All patients with AL and pelvic collection clinically manifested as abnormal drainage and radiological changes. Antibiotics and prolonged drainage were used for 10 of them (Clavien–Dindo grade less than  III). Two patients with severe AL (Clavien–Dindo grade greater or equal to III) treated by reoperation.

### Expression profile of IFs in abdominal drainage after colorectal surgery

**Fig. 1:**
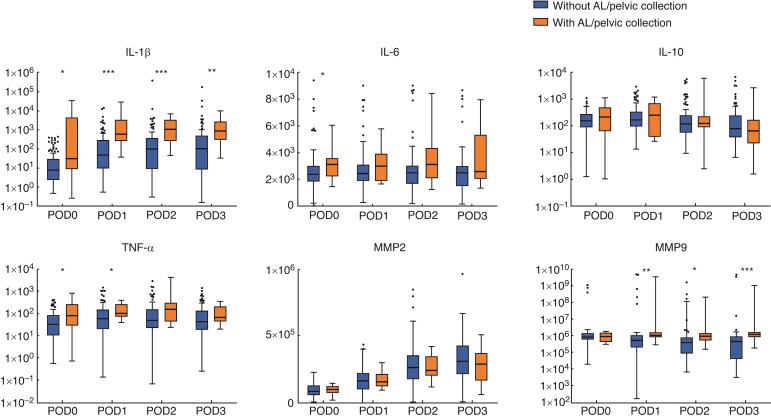
**Expression profiles of inflammatory factors in drain fluid between patients with or without anastomotic leakage/pelvic collection** Compared with patients without AL/pelvic collection, most intra-abdominal IFs in patients with AL/pelvic collection were obviously elevated at an early stage after surgery. Particularly, on POD 1, the contents of IL-1β, TNF-α, and MMP9 in patients with AL/pelvic collection are remarkably higher than those in patients without AL. *P < 0.05. **P < 0.01. ***P < 0.001. IF, inflammatory factors; AL, anastomotic leakage; POD, postoperative day; IL, interleukin; TNF, tumour necrosis factor; MMP, matrix metalloproteinase.

Drain fluid IF levels in the drain fluid are presented in *Fig. 1* and *Table S1*. Probably as expected, when compared with patients without AL and pelvic collection, most intra-abdominal IFs in patients with AL and pelvic collection were elevated at an early stage after surgery. The levels of IL-1β, TNF-α, and MMP9 in patients who developed AL and pelvic collection were much higher than those in other patients since POD1. Due to the possible influence of surgical procedure on drainage IFs, the IF levels and dynamic changes between the open group and laparoscopic group were compared (*Fig. S1* and *Table S2*). Most IFs between two groups within the first three PODs were not statistically different, except for IL-1β in POD1–2, IL-10, and TNF-α on POD1.

### AL and pelvic collection prediction models

By measuring serum CRP and drainage MMP9 on POD3, the individual risk for AL and pelvic collection were calculated. ROC analysis showed that the AUC of the prediction model was 0.77, which was very similar to its reported accuracy (0.78) (*Fig. S2*)^10^.

**Fig. 2:**
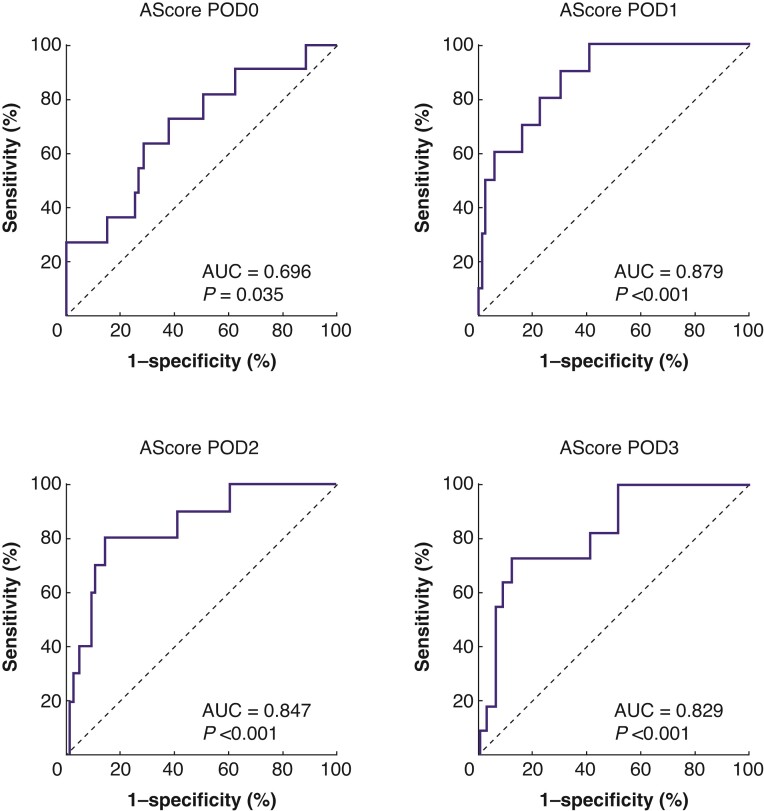
**Receiver operating characteristic analysis of AScore POD0, POD1, POD2, and POD3** ROC analysis showed that AScore at POD1 achieved the best diagnostic efficiency (AUC 0.879; 95% c.i., 0.64 to 0.89). ROC, receiver operating characteristic; POD, postoperative day; AUC, under the curve.

**Fig. 3:**
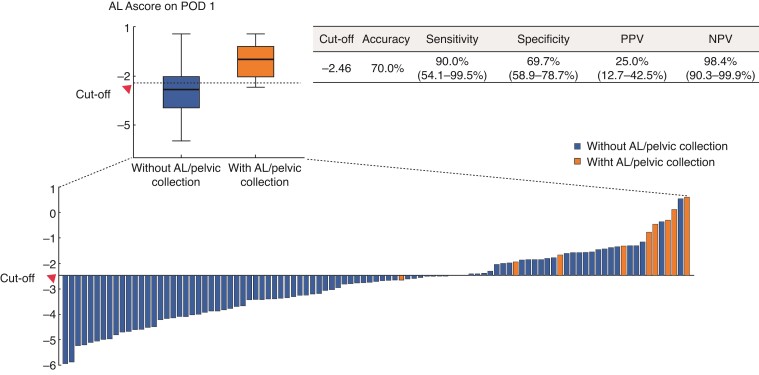
**Diagnostic efficacy of AScore at postoperative day 1** Orange box and bars indicate AScore at POD1 of patients with AL/pelvic collection. Blue box and bars indicate AScore at POD3 of patients without AL/pelvic collection. The maximum Youden index (sensitivity + specificity − 1) was −2.46, which was set as the cut-off value. The histogram represents the distribution of the AScore at POD1 among patients with or without AL/pelvic collection. Orange columns indicate patients with AL/pelvic collection. Blue columns indicate patients without AL/pelvic collection. Patients with AScore at POD1 higher or lower than −2.46 were classified into a high-risk or low-risk group respectively. AL, anastomotic leakage; POD, postoperative day; PPV, positive predictive value; NPV, negative predictive value.

To improve the diagnostic performance of IFs for AL and pelvic collection, the LASSO binary logistic regression was used to select the most predictive parameters out of numerous IFs and to establish the AScore systems. There were four AScore systems corresponding to different time points. The calculation formula of the AScore systems is shown in *Table 2*. Compared with Sparreboom's model, AScore on POD3 increased the AUC from 0.77 to 0.83. Moreover, AScore on POD1 showed the best diagnostic efficiency, with an AUC of 0.88 (*Fig. 2* and *Table 3*). Furthermore, AScore at POD1 still performed well in subgroup analysis by patient sex, age, BMI, or surgical procedure (*Table S3*). According to the Youden index, the cut-off value of AScore on POD1 was set as −2.46. Patients were then stratified into low-risk group and high-risk group by this cut-off. The distribution of AScore at POD1 among patients with or without AL or pelvic collection is shown in *Fig. 3*. The predictive model achieved 90.0 (54.1 to 99.5) per cent sensitivity, 69.7 (58.9 to 78.7) per cent specificity, 25.0 (12.7 to 42.5) per cent per cent PPV, and 98.4 (90.3 to 99.9) per cent NPV.

**Table 2:** Calculation formula of the AScore systems

	Formula
**POD0**	−4.15 + 0.64 × log_10_(**IL-1β^POD0^**) + 0.72 × log_10_(**IL**-**6^POD0^**) − 0.53 × log_10_(**IL**-**10^POD0^**)
**POD1**	−11.56 + 1.36 × log_10_(**IL**-**1β^POD1^**) − 1.22 × log_10_(**IL-10^POD1^**) + 0.28 × log_10_(**TNF**-**α^POD1^**) + 1.65 × log_10_(**MMP2^POD1^**) + 0.0073 × log_10_(**MMP9^POD1^**)
**POD2**	−7.85 + 0.84 × log_10_(**IL**-**1β^POD2^**) + 0.57 × log_10_(**IL**-**6^POD2^**) − 0.79 × log_10_(**IL**-**10^POD2^**) + 0.50 × log_10_(**TNF**-**α^POD2^**) + 0.45 × log_10_(**MMP2^POD2^**) + 0.03 × log_10_(**MMP9^POD2^**)
**POD3**	−6.32 + 0.54 × log_10_(**IL**-**1β^POD3^**) + 0.85 × log_10_(**IL**-**6^POD3^**) −0.79 × log_10_(**IL**-**10^POD3^**) + 0.06×log_10_(**TNF**-**α^POD3^**) + 0.30×log_10_(**MMP9^POD3^**)

IL, interleukin; MMP, matrix metalloproteinase; POD, postoperative day.

**Table 3:** Receiver operating characteristic analysis of the AScore systems

AScore systems	AUC	*P*	95% c.i.
**AScore POD0**	0.70	0.04	0.53–0.86
**AScore POD1**	0.88	<0.001	0.78–0.97
**AScore POD2**	0.85	<0.001	0.72–0.97
**AScore POD3**	0.83	<0.001	0.70–0.96

POD, postoperative day; AUC, the area under the curve.

## Discussion

This prospective cohort study investigated the feasibility of diagnosing AL following anastomosis and pelvic collection following APR by detecting abdominal drainage after colorectal surgery. The Sparreboom's prediction model was first validated, which showed that for patients with colorectal cancer, serum CRP, and peritoneal MMP9 is also reliable for diagnosing AL on POD3. A new prediction model was subsequently established by combining MMP9 and other IFs (such as IL-1β, TNF-α, and MMP2) in abdominal drainage, which achieved higher predictive efficacy in the very early postoperative interval (POD1). This study provided a new tool to predict AL and pelvic collection in POD1 to identify patients at low risk for these outcomes.

For patients who underwent colorectal resection, CRP is routinely tested to assist with the diagnosis of postoperative infectious complications or inflammation. However, predicting AL by CRP in the early postoperative days is not as accurate as expected. For instance, the PREDICT study, which was conducted among large multicentre samples, has shown that neither CRP trajectory nor daily values could meet the requirement of an AUC exceeding 0.80^9^. Because elevated serum CRP is mainly stirred by systemic inflammatory response, local infections such as asymptomatic AL are often accompanied by normal CRP levels at an early stage. Only when the complication advances to a systematic level, could it be detected by CRP or other systematic inflammatory parameters. In contrast, IF concentration in drainage is easily influenced by inflammation within the intra-abdominal microenvironment. Some research confirmed that local biomarkers from peritoneal fluid were more specific than systemic biomarkers^16^.

Sparreboom and colleagues were the first to investigate the diagnostic value of peritoneal IFs with serum CRP to predict AL in European patients undergoing rectal surgery. Their results showed that peritoneal MMPs improved the diagnostic value in detection of AL over serum CRP alone^10^. This study validated their model in a Chinese population and the results provide solid support to Sparreboom and colleague’s model in the diagnoses of AL and pelvic collection on POD3 in a general patient population. These results also demonstrate that clinical data registration and laboratory methodology of this research reached very good consistency with the APPEAL study. Nevertheless, Sparreboom's model can only be applied on the third day after surgery while the drainage tube is often removed. The placement of drainage systems might impair patient mobilization; hence the use of intra-abdominal/pelvic drainage is left at the surgeon’s discretion in ERAS in protocols^17,18^. The advantage of the AScore system is further emphasized, as it shortens the diagnosis time of AL and pelvic collection. The cut-off value was set as −2.46 on POD1, which yielded 90 per cent sensitivity. This means that 90 per cent of those who would develop AL later, were identified through the model. Moreover, the AScore system could be a valuable screening tool in the context of the growing popularity of ERAS protocols.

The selection of the corresponding IFs and their diagnostic value on POD1 is based on the early pathological changes of wound healing. In the cohort, the concentration of IFs in peritoneal fluid was obviously different between patients with and without AL and pelvic collection. According to previous studies^19,20^, excessive infiltration of immune cells and increased IF production during the early postoperative phase could disrupt physical wound healing. For AScore on POD1, it included five parameters such as IL-1β, IL-10, TNF-α, MMP2, and MMP9, all of which were major effect molecules involving inflammatory response and tissue repair. For instance, overexpressed IL-1β can reduce blood supply via interfering microvessel formation^21,22^. IL-10 reduces scar formation but delays the healing process^23^. TNF-α prolongs inflammation and impairs the healing response^24^. MMPs degrade extracellular matrix (ECM) proteins, especially collagen, and seriously weakens the strength of anastomosis^25,26^. Given the complex regulation system, the diagnostic value of individual IFs was unsatisfactory^27–29^. The LASSO regression was implemented for selecting the most useful parameters out of numerous variables to construct a regression model, avoiding overfitting^30^. The good diagnostic value confirms the diagnostic efficacy of IFs. A similar AL predicting model after gastrectomy was previously established and validated with the same method^31^. There were several limitations in this study. First, this work was performed in a single medical centre, and the cohort volume was not large enough to validate the reliability of AScore systems internally and externally. Second, the present study was observational, and the causal relationship between IFs and AL needs to be further explored, which might provide potential therapeutic targets for AL. Finally, this research did not analyse cost-effectiveness of the new diagnostic method. As the measurement of IFs is not a routine test item in clinical practice, future study should focus on optimizing the methodology and reducing test costs.

## Supplementary Material

zrac069_Supplementary_Data

## Data Availability

The data sets generated and analysed during the present study are available from the corresponding author on reasonable request.
