# Supplementary material for: Early diagnosis of anastomotic leakage after colorectal cancer surgery using an inflammatory factors-based score system
Source: BJS Open. 2022 Jun 3;6(3):zrac069. doi: 10.1093/bjsopen/zrac069 (PMC9165091; doi:10.1093/bjsopen/zrac069)
Supplement: zrac069_Supplementary_Data [file zrac069_supplementary_data.zip › Supplementary_material.docx]

#### Supplementary material

**Early Diagnosis of Anastomotic Leakage after Colorectal Cancer Surgery via an Inflammatory Factors Based Score System**

**Additional file:**

**Table S1.** Quantitative Analysis of IFs in Drain Fluid Between Patients with or without AL/pelvic collection

**Table S2.** Quantitative Analysis of IFs in Drain Fluid Between Open and Laparoscopic Group

**Table S3.** The Stratified ROC Analysis of the AScore-POD1

**Figure S1.** The trajectories of IFs in Drain Fluid after surgery

**Figure S2.** ROC analysis of Sparreboom’s AL prediction model

**Table S1. Quantitative Analysis of IFs in Drain Fluid Between Patients with or without AL**

| **IFs** | **POD** | **Without AL/pelvic collection** (pg/mL) |  | **With AL/pelvic collection** (pg/mL) | ***P**** |
| --- | --- | --- | --- | --- | --- |
| **IL-1β, median (IQR)** | POD 0 | 6.160 (1.79-16.67) |  | 31.61(17.60-8548.87) | 0.048 |
|  | POD 1 | 37.98 (2.54-148.65) |  | 582.81 (332.74-3475.00) | 0.001 |
|  | POD 2 | 93.31 (7.48-338.56) |  | 1116.06 (316.36-3962.75) | 0.001 |
|  | POD 3 | 96.39 (5.41-458.50) |  | 924.71 (125.19-2421.50) | 0.003 |
|  |  |  |  |  |  |
| **IL-6, median (IQR)** | POD 0 | 2407.00 (1829.00-3052.00) |  | 3140.00 (1685.25-3200.00) | 0.049 |
|  | POD 1 | 2417.00 (1883.00-3090.00) |  | 2908.50 (1761.75-3339.00) | 0.187 |
|  | POD 2 | 2525.00 (1714.00-3090.00) |  | 3019.50 (1855.50-3212.25) | 0.073 |
|  | POD 3 | 2282.00 (1032.00-2976.00) |  | 2370.50 (1681.00-3162.50) | 0.095 |
|  |  |  |  |  |  |
| **IL-10, median (IQR)** | POD 0 | 147.75 (80.56-253.92) |  | 245.96 (63.83-611.53) | 0.611 |
|  | POD 1 | 135.49 (87.85-289.16) |  | 422.47 (55.33-896.43) | 0.781 |
|  | POD 2 | 118.97 (54.55-248.48) |  | 140.63 (99.13-238.12) | 0.593 |
|  | POD 3 | 69.23 (27.87-243.16) |  | 50.09 (23.51-181.54) | 0.452 |
|  |  |  |  |  |  |
| **TNF-α, median (IQR)** | POD 0 | 21.55 (6.99-67.91) |  | 81.07 (35.42-232.96) | 0.033 |
|  | POD 1 | 41.27 (14.28-114.22) |  | 151.93 (83.92-301.93) | 0.038 |
|  | POD 2 | 46.99 (18.92-154.68) |  | 253.02 (49.99-371.81) | 0.086 |
|  | POD 3 | 42.17 (13.09-130.07) |  | 51.25 (30.47-170.36) | 0.163 |
|  |  |  |  |  |  |
| **MMP-2, median (IQR)** | POD 0 | 87945.00 (53458.00-136243.00) |  | 107174.00 (71931.25-121117.25) | 0.790 |
|  | POD 1 | 176254.00 (102638.00-238014.00) |  | 173312.00 (130578.50-269136.50) | 0.724 |
|  | POD 2 | 263835.00 (183983.00-353238.00) |  | 242170.50 (224159.50-320116.75) | 0.962 |
|  | POD 3 | 307912.00 (211066.00-426651.00) |  | 287523.00 (215925.50-353523.50) | 0.633 |
|  |  |  |  |  |  |
| **MMP-9, median (IQR)** | POD 0 | 821155.00 (490972.00-1207500.00) |  | 967835.50 (457092.50-1462867.25) | 0.769 |
|  | POD 1 | 384686.00 (122365.00-1747347.00) |  | 1048489.00 (733438.50-1501589.00) | 0.007 |
|  | POD 2 | 285939.00 (69445.00-667650.00) |  | 938137.00 (489777.75-1237808.50) | 0.014 |
|  | POD 3 | 297403.00 (33195.00-789827.00) |  | 1284666.50 (931680.00-1471819.00) | <0.001 |

Abbreviations: IFs, inflammatory factors; AL, anastomotic leakage; IL-1β, interleukin-1β; IL-6, interleukin-6; IL-10, interleukin-10; TNF-α, tumor necrosis factor-α; MMP2, metalloproteinase2; MMP9, metalloproteinase9; IQR, interquartile range; POD, postoperative day. * Mann–Whitney tests.

**Table S2. Quantitative Analysis of IFs in Drain Fluid Between open and Laparoscopic group**

| **IFs** | **POD** | **Open** |  | **Laparoscopic** | ***P**** |
| --- | --- | --- | --- | --- | --- |
| **IL-1β, median (IQR)** | POD 0 | 7.42 (2.26-43.19) |  | 7.32(1.38-26.04) | 0.828 |
|  | POD 1 | 5.97 (1.40-108.00) |  | 108.75 (19.34-291.13) | 0.016 |
|  | POD 2 | 23.31 (1.26-253.90) |  | 139.30 (42.93-494.70) | 0.041 |
|  | POD 3 | 26.06 (1.25-530.61) |  | 167.55 (28.20-634.97) | 0.124 |
|  |  |  |  |  |  |
| **IL-6, median (IQR)** | POD 0 | 2376.00 (1773.50-2995.50) |  | 2471.00 (1839.75-3121.50) | 0.615 |
|  | POD 1 | 2325.50 (1824.50-2784.00) |  | 2735.50 (1869.50-3176.25) | 0.138 |
|  | POD 2 | 2400.50 (1760.00-2952.50) |  | 2635.50 (1721.75-3159.50) | 0.705 |
|  | POD 3 | 2041.50 (988.61-2812.25) |  | 2550.00 (1670.50-3100.75) | 0.861 |
|  |  |  |  |  |  |
| **IL-10, median (IQR)** | POD 0 | 171.94 (110.46-308.80) |  | 136.97 (66.29-251.23) | 0.471 |
|  | POD 1 | 117.14 (58.06-266.90) |  | 156.05 (92.24-378.97) | 0.033 |
|  | POD 2 | 77.60 (34.88-208.28) |  | 126.96 (60.60-247.15) | 0.090 |
|  | POD 3 | 54.57 (27.13-175.33) |  | 85.17 (33.87-247.15) | 0.301 |
|  |  |  |  |  |  |
| **TNF-α, median (IQR)** | POD 0 | 22.27 (9.36-82.32) |  | 28.42 (7.32-71.32) | 0.962 |
|  | POD 1 | 23.50 (8.84-45.01) |  | 75.68 (36.24-191.06) | <0.01 |
|  | POD 2 | 40.94 (19.35-229.68) |  | 55.59 (25.08-189.11) | 0.464 |
|  | POD 3 | 41.28 (12.82-186.25) |  | 51.25 (19.45-121.23) | 0.909 |
|  |  |  |  |  |  |
| **MMP-2, median (IQR)** | POD 0 | 86461.00 (53458.00-137417.00) |  | 87945.00 (54392.00-127844.50) | 0.788 |
|  | POD 1 | 179313.00 (102638.00-273050.00) |  | 172552.00 (115095.50-230989.50) | 0.401 |
|  | POD 2 | 270726.50 (214720.00-375111.00) |  | 258255.50 (197088.50-324185.00) | 0.222 |
|  | POD 3 | 372125.00 (242135.00-464888.00) |  | 302716.50 (203972.00-385122.50) | 0.275 |
|  |  |  |  |  |  |
| **MMP-9, median (IQR)** | POD 0 | 837897.00 (435551.00-922337.20) |  | 852109.00 (513320.00-1193991.00) | 0.792 |
|  | POD 1 | 190303.50 (81047.00-922337.20) |  | 524244.50 (235837.50-824977.00) | 0.201 |
|  | POD 2 | 144679.00 (52767.00-543492.00) |  | 480762.00 (174694.00-863832.00) | 0.111 |
|  | POD 3 | 73158.50 (27959.00-757880.00) |  | 486046.50 (137543.00-922337.20) | 0.284 |

Abbreviations: IFs, inflammatory factors; AL, anastomotic leakage; IL-1β, interleukin-1β; IL-6, interleukin-6; IL-10, interleukin-10; TNF-α, tumor necrosis factor-α; MMP2, metalloproteinase2; MMP9, metalloproteinase9; IQR, interquartile range; POD, postoperative day. * Mann–Whitney tests.

**Table S3. The Stratified ROC Analysis of the AScore-POD1**

| **Characteristic** | | **AUC** | **95%CI** | ***P*** |
| --- | --- | --- | --- | --- |
| **Gender** |  |  |  |  |
|  | Male | 0.926 | 0.82-1.00 | <.001 |
|  | Female | 0.855 | 0.72-0.99 | <.001 |
| **Age** |  |  |  |  |
|  | ＜65 | 0.88 | 0.78-0.99 | <.001 |
|  | ≥65 | 0.88 | 0.67-1.00 | <.001 |
| **Body mass index** |  |  |  |  |
|  | ＜24 | 0.95 | 0.86-1.00 | <.001 |
|  | ≥24 | 0.86 | 0.73-0.99 | <.001 |
| **Surgical Procedure** |  |  |  |  |
|  | Open | 0.90 | 0.75-1.00 | <.001 |
|  | Laparoscopic | 0.87 | 0.76-0.99 | <.001 |
| **Tumor Location** |  |  |  |  |
|  | Colon | 0.96 | 0.90-1.00 | <.001 |
|  | Rectal | 0.84 | 0.71-0.97 | <.001 |

Abbreviations: ROC, receiver operating characteristic analysis; AScore, anastomotic leakage score; POD, postoperative day; AUC, the area under the curve.


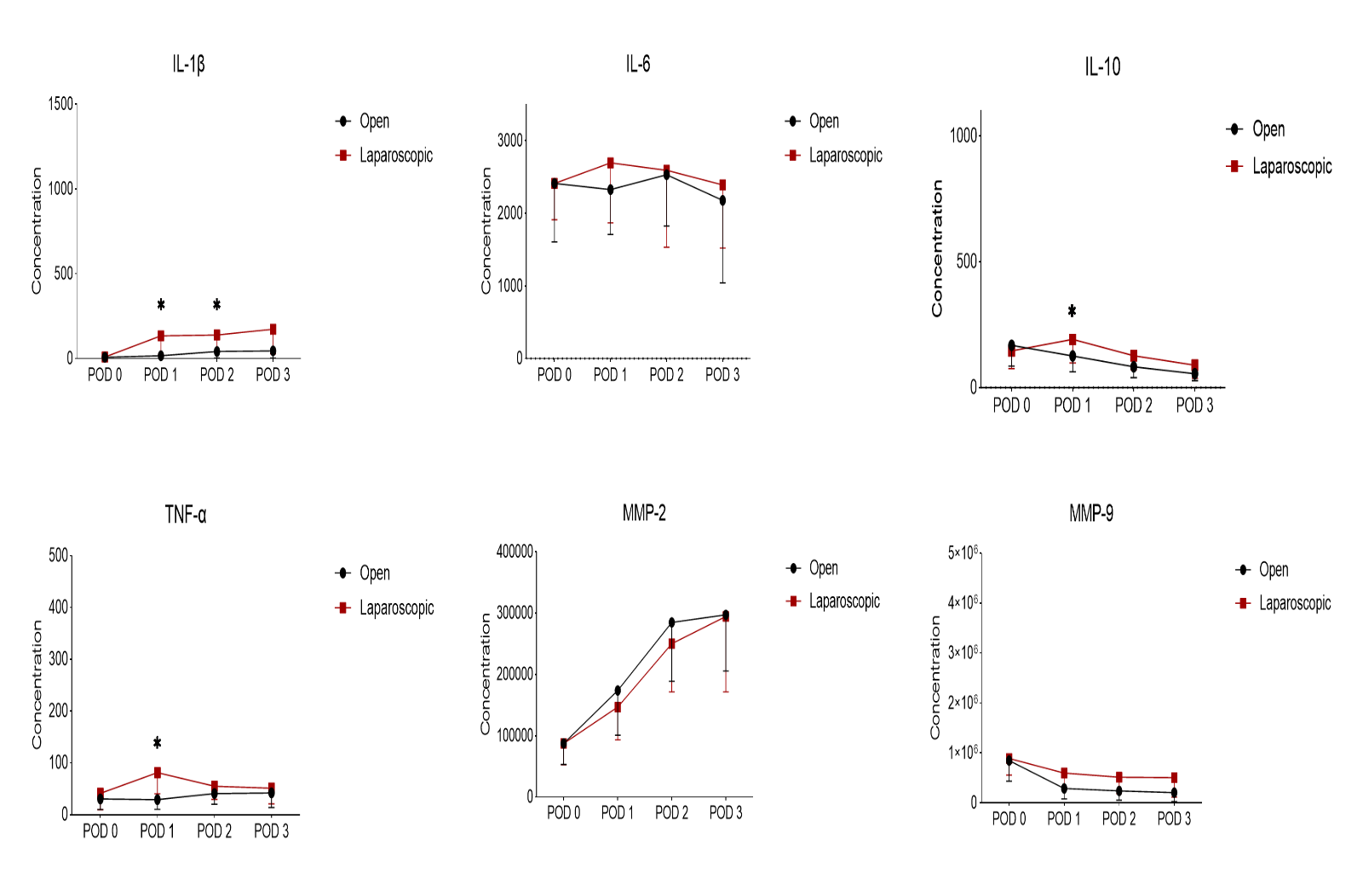
**Figure S1. The trajectories of IFs in Drain Fluid after surgery**

Mann–Whitney tests. * < 0.05, ** < 0.01, *** < 0.001

**Figure S2. ROC analysis of Sparreboom’s prediction model**

**
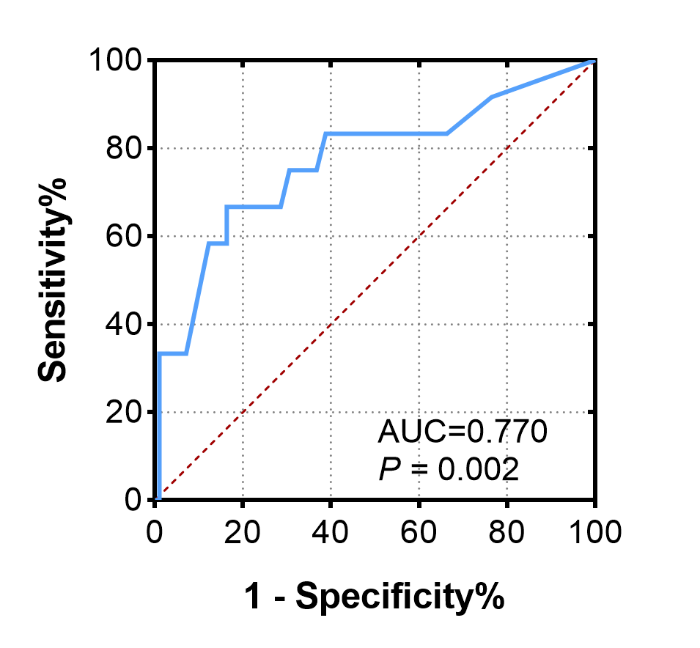
**
